# Multidisciplinary approach in diagnosis and treatment of COVID-19-associated mucormycosis: a description of current reports

**DOI:** 10.1186/s43162-022-00143-7

**Published:** 2022-07-23

**Authors:** Hyma Gogineni, Wonhee So, Kenneth Mata, John N. Greene

**Affiliations:** 1 Western University of Health Sciences, 309 E. 2nd street, Pomona, CA 91766 USA; 2 Moffitt Cancer Center, Tampa, FL USA; 3 Department of Internal Medicine, Morsani College of Medicine, University of South Florida, Tampa, FL USA

**Keywords:** COVID-19, Mucormycosis, Multidisciplinary team, CAM

## Abstract

**Background:**

We reviewed the epidemiology, risk factors, pathophysiology, and clinical presentations of coronavirus disease 2019 (COVID-19)-associated mucormycosis (CAM), then discussed the importance of rapid diagnosis and treatment facilitated by multidisciplinary approach.

**Main body:**

India has reported world’s highest number of CAM cases where *Rhizopus arrhizus* was the most predominant etiology. CAM caused by *Rhizopus microsporus* was the most common from the rest of the world. Multiple risk factors for CAM were identified including diabetes mellitus, inappropriate corticosteroid use, COVID-19-related hypoxia, and lung damage.

Rhino-orbito-cerebral mucormycosis (ROCM) accounted for almost 90% of CAM in India while 64% of global cases were ROCM. Less than 10% of CAM from India were pulmonary while the rest of the world reported 21% of pulmonary CAM.

CAM is diagnosed by confirmed SARS-CoV2 infection along with clinical, radiological, histopathological, and/or microbiological evidence of mucormycosis. In patients with risks of CAM and associated symptoms, CT or MRI are recommended. If ROCM is suspected, endoscopy and biopsy are recommended. If pulmonary CAM is suspected, tissue biopsies, nasal samples, or bronchoalveolar lavage is recommended with histopathological exams.

Early diagnosis, surgical, and pharmaceutical interventions are key to treat mucormycosis. Upon diagnosis, antifungal therapy with liposomal amphotericin B (IV) is considered first-line of therapy. Alternatively, posaconazole (PO/IV) or isavuconazole (PO/IV) can be used.

**Conclusion:**

Treating CAM requires a multidisciplinary approach for early diagnosis and prompt initiation of interventions to maximize patient’s chance of survival.

## Background

As coronavirus disease 2019 (COVID-19) pandemic continues to pose an ongoing public health threat, a myriad of complications have been reported including secondary bacterial [1] and invasive fungal infections [2]. While mucormycosis is considered a rare invasive fungal infection that usually occurs in highly immunocompromised hosts, a recent unprecedented surge of COVID-19-associated mucormycosis (CAM) in India was reported in patients with diabetes, high doses of steroid use, and frequent use of antibiotics [3, 4]. This drew a global attention and Indian health authorities have made it a notifiable disease [3, 4]. In India, at least 45,374 cases of CAM and more than 4300 deaths have been reported as of July 21, 2021 [5].

Popularly known as the “Black Fungus” infection, mucormycosis is rapidly fatal if left untreated. Early recognition of the disease and prompt initiation of treatment are essential for lifesaving management. Due to the challenges and complexity associated with early diagnosis and management of CAM, it would be best handled by a multidisciplinary team. In this review, we intended to improve clinician’s understanding of epidemiology, risk factors, pathophysiology, and clinical features of CAM and to ultimately prompt multidisciplinary team approach in diagnosis and management of CAM.

## Main Text

### Epidemiology

Mucormycosis is an invasive fungal infection caused by a group of molds that belong to the order of Mucorales. The most common species associated with mucormycosis are *Rhizopus* spp., *Mucor* spp., and *Lichtheimia* spp. followed by *Rhizomucor* spp., *Cunninghamella* spp., *Apophysomyces* spp., and *Saksenaea* spp [6, 7]. An epidemiology study from 2010 to 2014 reported *Rhizopus arrhizus* as the most common etiology of mucormycosis in India [8]. Consistently, *Rhizopus arrhizus* was the most predominant etiology causing CAM in India [9] while *Rhizopus microsporus* was most common in CAM cases from the rest of the world [10]. Interestingly, six out of the seven CAM cases caused by *R. microsporus* were pulmonary and the last one was a cutaneous mucormycosis [10].

Due to its rarity, lack of surveillance, and difficulty in diagnosis, the incidence rate of mucormycosis is hard to measure. Nonetheless, according to the World Health Organization (WHO), the incidence rate of mucormycosis globally varies from 0.005 to 1.7 per million population [3]. India reports the world’s highest prevalence of mucormycosis of 140 per million population, which is 80 times higher than that of developed countries [3]. Overall incidence of mucormycosis had been on the rise prior to COVID-19 pandemic [11, 12]. For example, the USA reported increased rate of mucormycosis from 0.12 in 2005 to 0.16 per 100,000 patients in 2014 [13, 14]. Given the highest prevalence in India prior to the pandemic, it is not surprising that India has the highest rates of CAM. In a recent systematic review, 101 cases of CAM were reported worldwide, of which 82 cases (81%) were from India, 9 cases from the USA, and 3 cases from Iran [15].

### Risk factors

In developed countries, the rise of mucormycosis is linked to the increasing patient population with hematological malignancies undergoing chemotherapy or cancer immunotherapy, solid organ, and hematopoietic stem transplantation [11–13]. On the other hand, in developing countries such as in India, diabetes mellitus is the most predominant risk factor for mucormycosis, followed by hematological malignancies and transplantations [11]. Of note, China hardly has any reported cases of CAM in contrast to India despite having world’s largest diabetic population, which indicate the contributors to outbreak for CAM might be multifactorial [16]. Other risk factors for mucormycosis in India include history of prolonged use of corticosteroids, iron overload, auto-immune disease, chronic kidney disease, pulmonary tuberculosis, and chronic obstructive pulmonary disease [17]. A multicenter study in India by Patel et al. identified newly detected diabetes as a significantly more frequent condition among CAM (29/187 [21%]) than among non-CAM (10/100 [10%]; *p* = 0.02) patients [9]. Notably, COVID-19 was the only underlying condition in 61/187 (33%) of CAM patients [9]. Authors also found inappropriate use of corticosteroid (i.e., higher dose or longer duration than recommended regimen, inappropriately given when patients were not hypoxic) and COVID-19-related hypoxia to be independent risk factors for CAM [9]. Interestingly, the use of corticosteroid was not an independent risk factor for COVID-19-associated pulmonary aspergillosis [2] but has been identified as a risk factor for CAM in several studies [9, 17, 18].

There is also an environmental study that showed high numbers of Mucorales spores both outdoor and inside hospitals in Northern India [19]. Along with the environmental factors and abovementioned risk factors, the endothelial damage caused by SARS-CoV2, intracellular iron overload driven by escalated ferritin level in hyperinflammatory stage of severe COVID-19, and overexpression of the glucose regulated protein (GRP78) have also been suggested to be the contributors to the current outbreak of CAM [4].

### Pathophysiology

Initial infection occurs mainly via inhalation of spores but also by direct inoculation or digestion of fungal spores from decaying organic matter (i.e., soil, leaves, rotten wood) [16, 17]. Once inside the body, fungal spores then swell, germinate, and produce fast-growing hyphae, which then causes blood vessel penetration, blood clotting, and tissue necrosis [20]. The organism can disseminate from the original site of infection to other sites of the body [16, 17].

### Clinical presentation

Rhino-orbito-cerebral mucormycosis (ROCM) initially starts in the paranasal sinuses but can extend to the orbit and occasionally to the brain [21–23]. Early signs include facial pain, nasal blockage or congestion, and bloody/brown/black discharge, with or without local pain. Nasal ulcers or crusts can form later. Facial edema, palatal ulcer leading to a dark necrotic area, toothache, and maxillary pain can also be seen. Orbital invasion could result in blurry vision, orbital pain, ophthalmoplegia, and proptosis. Cranial nerve palsies are common and cerebral invasion can cause cerebral edema, thrombosis, and infarcts [4]. During the current CAM epidemic, ROCM accounted for almost 90% of cases in India while only 64% of global cases were ROCM [10]; this is not surprising given ROCM usually occurs in diabetics whereas such patients rarely present with pulmonary infection [24].

Pulmonary mucormycosis can present with fever, cough, hemoptysis, pleuritic chest pain, pleural effusions, and tissue infarction [9]. This form of mucormycosis is the most common in neutropenic patients. Less than 10% of CAM from India were pulmonary while 21% of CAM were pulmonary from the rest of the world [10].

Cutaneous mucormycosis mostly affects arms and legs [25]. It initially starts as erythematous lesions but can turn purple, and eventually black. It is challenging to differentiate cutaneous mucormycosis from aspergillosis or synergistic gangrene caused by bacterial infections. In general, cutaneous mucormycosis is seen in trauma patients [26] while it is scarcely reported in CAM patients [13].

Gastrointestinal mucormycosis most frequently involves the stomach, followed by the colon and ileum. It is the rare form of mucormycosis that is caused by ingestion of fungal spores. Patient presents with unspecific gastrointestinal symptoms such as fever, gastric pain, gastrointestinal bleeding, acute diarrhea, abdominal distension, and colonic perforation [7]. While it is rare, there are case reports of gastrointestinal CAM both in immunocompromised and immunocompetent patients [27–29].

Disseminated mucormycosis is when the infection spreads from one organ to another. Most common sites are lungs, sinus, soft tissues, central nervous system, liver, and kidney [3, 30]. While mortality from mucormycosis ranges from 40 to 80%, the disseminated disease especially to the brain has the highest mortality rate over 80% [7].

### Diagnosis

Detailed guidelines on diagnosis of mucormycosis have been published [7]. Patients with suspected CAM should be referred immediately to a facility with the highest level of care [7]. For diagnosis of CAM, SARS-CoV-2 infection should be confirmed by RT PCR test of RNA or antigen testing along with clinical, radiological, histopathological, or microbiological evidence suggestive of mucormycosis [4].

#### Imaging

**Fig. 1:**
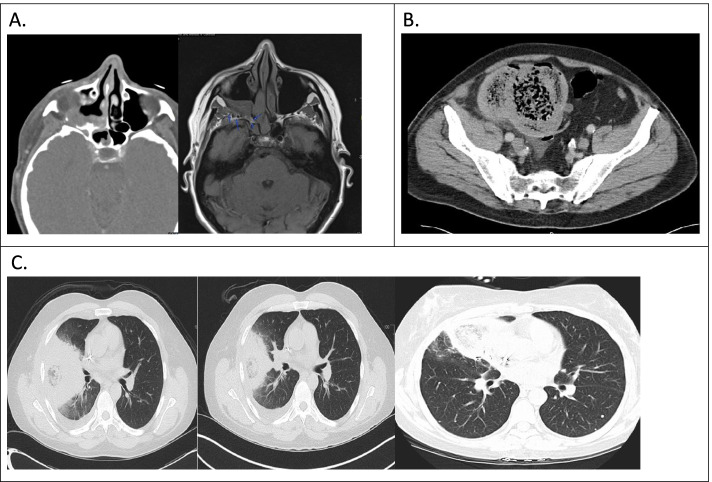
Radiographic signs of mucormycosis. **A** Maxillary sinus mucormycosis. **B** Gastrointestinal mucormycosis of cecum. **C** Pulmonary mucormycosis: reversed halo sign on CT, an area of ground glass opacity surrounded by a ring of consolidation

In diabetic patients with facial pain, sinusitis, proptosis, or ophthalmoplegia, computed tomography (CT) or magnetic resonance imaging (MRI) of the brain and paranasal sinuses are recommended to see if sinusitis is present [7]. MRI is better than CT in determining the extent of fungal tissue invasion thus preferred when suspecting eye or brain disease [7]. Radiological findings of ROCM include mucosal thickening, opacification of sinuses, edema, inflammation, or infarction of the brain [4]. If sinusitis is diagnosed, endoscopy and biopsy are recommended to diagnose mucormycosis (Fig. 1) [7].

Radiological characteristics of pulmonary mucormycosis overlap with the findings from pulmonary aspergillosis, thus posing a diagnostic challenge. The reversed halo sign, an area of ground glass opacity encircled by consolidation on CT of thorax, or a thick-walled lung cavity and multiple nodules are commonly associated with pulmonary mucormycosis (Fig. 1) [7].

#### Mycological diagnosis

Because it is difficult to differentiate mucormycosis from aspergillosis radiographically, the definite diagnosis is done from the tissue biopsies and through microbiological and/or histopathological examinations. If biopsies are not feasible, nasal samples from ROCM or bronchoalveolar lavage (BAL) samples from pulmonary mucormycosis can be used for microbiological diagnosis.

Histopathologically, Mucorales are characterized by wide and irregular, ribbon-like hyphae [7]. Hyphae can be 6–16 μm wide, but may be up to 25 μm, and are non-septate or pauci­septate. Hyphae can confusingly look septated if tissue itself folds over during processing [7]. Similarly, the historically described 90° branching angle of Mucorales in tissue as compared to 45° branching angle of septated molds such as *Aspergillus*, can be difficult to identify due to changes in tissue architecture during processing [7]. Due to these challenges, it is important to confirm the diagnosis by cultures or other measures such as PCR and matrix-assisted laser desorption/ionization-time of flight (MALDI-TOF) [4]. With the histopathologic stain, Mucorales lesions show angioinvasion, necrosis, neutrophilic infiltration (in non-neutropenic patients), hemorrhagic infarction, and perineural invasion [4].

### Treatment

Early diagnosis, surgical and pharmaceutical interventions are key to treat mucormycosis. Also, it is critical to control underlying predisposing factors by proper glycemic control, judicious use of corticosteroid for the treatment of COVID-19, and adjustment of any immunosuppressants or immunomodulators as needed [31].

#### Surgical intervention

Early and aggressive surgical debridement is the core of mucormycosis therapy [31]. Local control of the disease with wide and repeated surgical debridement was associated with improved outcomes [32]. In ROCM, removal of the palate, nasal cartilage, and the orbit leads to disfigurement. Therefore, complete debridement of the external tissues and endoscopic debridement of internal tissues are more optimal. In pulmonary mucormycosis, surgical treatment also has shown to significantly improve survival when used in combination of antifungal treatment [31].

#### Antifungal pharmacotherapy

**Fig. 2:**
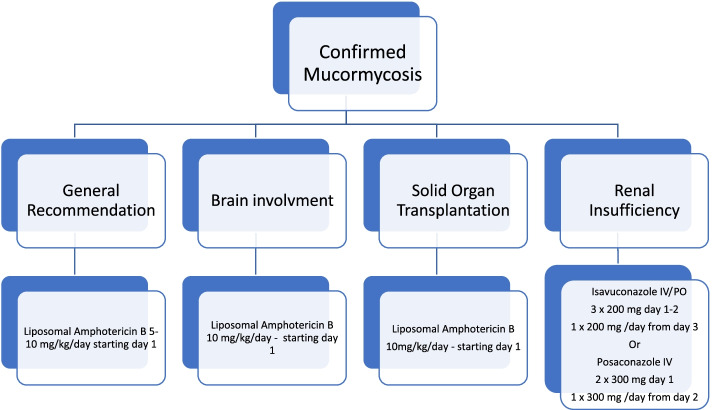
Guideline recommended pharmacotherapy [7]

Upon diagnosis of mucormycosis, antifungal therapy with Amphotericin B is considered first-line and should be initiated promptly (Fig. 2). Efficacy of amphotericin B has been shown in both in vitro, in vivo, and clinical studies [31]. The conventional amphotericin B deoxycholate (AmBD) has different dosing recommendations from its three lipid formulations and has largely been replaced with the lipid formulations in clinical use. The lipid formulations were designed to reduce nephrotoxicity of AmBD and have shown to be less nephrotoxic in actual clinical use: amphotericin B lipid complex (Abelcet, ABLC), amphotericin B cholesterol sulfate or amphotericin B colloidal dispersion (Amphotec, ABCD), and liposomal amphotericin B (Ambisome, L-AmB) [33]. Even though lipid formulations of amphotericin B are less nephrotoxic, electrolytes, particularly potassium and magnesium, should still be monitored [34].

If the patient cannot tolerate amphotericin B or the drug is not available, posaconazole or isavuconazole can be used [35, 36]. Posaconazole originally became available as an immediate release (IR) oral suspension, which had several pharmacokinetic limitations. For example, posaconazole oral suspension needs to be taken with a fatty meal to increase absorption and requires low gastric pH. Later, posaconazole delayed release (DR) tablet and an intravenous (IV) formulation were developed with improved pharmacokinetic profile and eliminated the need for fatty meal and low gastric pH for adequate absorption [37]. Therefore, DR tablet is preferred to IR formulations as oral options. While it is controversial whether DR tablet or IV formulation still requires therapeutic drug monitoring (TDM), TDM is definitely recommended for posaconazole IR formulation [38]. In addition, since posaconazole is a strong CYP3A4 inhibitor, drug concentrations primarily metabolized by CYP3A4 needs to be monitored [39].

Isavuconazonium sulfate is available as both oral and IV formulations. Upon administration, it is rapidly cleaved into isavuconazole [36]. It is generally well tolerated with similar side effect profile to posaconazole. Unlike other azoles, isavuconazole does not cause QTc prolongation and might display QTc shortening; this makes it a viable option in a patient with prolonged QTc. Since isavuconazole is a mild to moderate inhibitor of CYP3A4, it is a useful alternative to posaconazole when patients are on essential medications that are CYP3A4 substrates [40]. In general, isavuconazole TDM is not necessary except when concerned for impaired drug absorption, therapeutic failure, or drug toxicity [41]. The monitoring of the efficacy and safety of these agents is crucial for treatment success (Table 1).

**Table 1:** Antifungal pharmacotherapy of mucormycosis [30–34]

	Drug name	Adverse effects	Monitoring parameters/caveats
**First line**	Amphotericin B lipid complex or liposomal amphotericin B	Infusion-related reactions, nephrotoxicity, electrolyte imbalance (hypomagnesemia, hypophosphatemia, hypokalemia, hypocalcemia), transaminitis	Renal function (SCr, BUN, urine output/input), electrolytes (potassium, magnesium, phosphorus), LFT, CBC, temperature
**Salvage therapy**	Posaconazole 300 mg IV/PO DR tablet every 12 h for the first day, then 300 mg IV/PO daily. IR oral suspension^a^ Posaconazole 200 mg PO q6h or 400 mg PO q12h	Diarrhea, nausea, vomiting, QTc prolongation, transaminitis	LFT, QTc, CBC, Posaconazole trough concentrations DR tablet can be taken with or without food; do not chew, divide, crush, or dissolve DR tablet ^a^IR suspension should be taken with a full meal and should be avoided with concurrent proton pump inhibitors
	Isavuconazonium sulfate 372 mg (isavuconazole 200 mg) IV/PO q8h × 6 doses, followed by 372 mg IV/PO or PO q24h thereafter	Nausea, vomiting, diarrhea, transaminitis, peripheral edema, back pain, QTc shortening	LFTs, QTc, isavuconazole trough concentration monitoring is not recommended except when concerned for impaired drug absorption, therapeutic failure, and toxicity

*SCr* serum creatinine, *BUN* blood urea nitrogen, *LFT* liver function tests, *CBC* complete blood count, *QTc* corrected QT interval, *DR* delayed release, *IR* immediate release, ^a^use only if posaconazole DR tablet is unavailable, *IV* Intravenous, *PO* oral

### Multidisciplinary approach

Suspected mucormycosis needs urgent intervention as delay in treatment is associated with increased mortality [25]. To maximize survival rates, multidisciplinary team approach for early recognition and treatment is essential including medical, surgical, radiological, laboratory-based teams, nurses, and pharmacists to name a few (Table 2). In the setting of pandemic, a case presentation and discussion can occur at daily multidisciplinary team rounds or via rapid communication between the teams arranged by multidisciplinary conference calls. All participants within the team are encouraged to participate in the discussion. The multidisciplinary team embraces an inclusive strategy and intends to make the care plan considering every team member’s input. The team not only makes the initial decision but also updates the members on patient’s progress. Multidisciplinary team’s discussion should include patient demographics, comorbidities, supporting system, preferences, clinical progress, and treatment recommendations [42, 43]. Patients along with their family members or caregivers should also be involved in the clinical decision-making process and be informed of the discussion from multidisciplinary team rounds.

**Table 2:** Multidisciplinary team member roles in caring for CAM patients

Team	Roles
Hospitalist, intensivist, primary care provider	1. Detect patients with early signs of mucormycosis 2. Consult specialists and services that should be part of the patient care 3. Initiate multidisciplinary team discussions 4. Serve as liaison between patient, patient caregivers, and the team
Ophthalmologist	1. Conducts eye examinations in suspicion of rhino-orbital-cerebral mucormycosis 2. Perform orbital surgical debridement when needed
Surgical specialist	1. Collect tissue for laboratory analyses 2. Perform surgical debridement when needed
Infectious disease specialist	1. Recommend appropriate diagnostic procedures 2. Assist in interpreting microbiology laboratory results 3. Review and revise therapy based on local epidemiology and susceptibility patterns 4. Select appropriate antifungal agents 5. Implement antifungal stewardship program to combat resistance
Clinical pharmacy specialist	1. Participate in multidisciplinary team discussions on antifungal drug selection 2. Recommend appropriate dose based on patient specific laboratory parameters 3. Monitor efficacy and adverse effects, therapeutic drug levels, and drug interactions 4. Recommend alternative therapies based on drug availability based on clinical practice guidelines 5. Educate patients and on antifungal therapies 6. Implement and monitor antifungal stewardship 7. Monitor drug costs by recommending formulary agents
Microbiologist	1. Promptly report critical results to the clinical care team - Fungal elements seen on microscopy - Immediate detection of fungal growth - Definite fungal identification 2. Ensure external and internal validation of mucormycosis 3. Discuss differential diagnosis and suggest additional testing when needed
Pathologist	1. Immediately report positive findings to the team 2. Discuss histopathology results with the team 3. Affirm quality control of fungal stains

#### Hospitalist

The hospitalist would serve as the primary provider for patients affected with CAM. Hospitalists must remain vigilant to recognize any early symptoms of mucormycosis. Upon high suspicion, the hospitalist should gather the clinical team of specialists, initiate a multidisciplinary discussion, and ensure diagnostic and therapeutic interventions are performed in a timely manner. They would serve as the liaison between the patient and the consulting teams. Because the hospitalist is acting as the patient’s representative and advocate, hospitalists should also relay the patient’s views and preferences during multidisciplinary team discussion.

#### Ophthalmologist

Ophthalmologists are needed to perform eye physical examinations upon suspicion of ROCM. Simple tests such as vision, pupil, ocular motility, and sinus tenderness can be part of routine physical evaluation [44]. Ophthalmologists can also order CT or MRI imaging to further investigate any suspicion of ROCM. They can also perform any orbital debridement as necessary.

#### Surgical specialists

Surgical specialists are essential in performing debridement, removing any necrotic tissue to stop the fungus from spreading throughout the body, and sending any tissue samples for laboratory analysis. Specialists may include neurosurgeons, otolaryngologists, or ophthalmologists. Surgical debridement is recommended, if feasible, for ROCM and for selected patients with localized pulmonary mucormycosis [42]. In ROCM, surgical debridement is performed primarily by otolaryngologists in collaboration with ophthalmologists and neurosurgeons for orbital and intracranial extension, respectively [32]. Depending on the severity of the fungal infection, repeated assessments and debridement are often required due to the aggressive nature of the infection [42, 45].

#### Infectious disease (ID) specialist

Consultation with an ID specialist has shown to improve the outcome of patients diagnosed with severe infections [42, 46, 47]. Considering the destructive and rapidly progressing nature of mucormycosis, ID specialist should be involved in every stage of the clinical decision-making process. The ID specialist can facilitate ordering the right diagnostic tests, selecting the appropriate antifungal regimen both for outpatient and inpatient settings. In addition, ID specialist should facilitate antifungal stewardship in the time of pandemic by promoting optimal use of diagnostics (e.g., ordering CT scans, fungal biomarkers, fungal stain), recommending most appropriate antifungal regimen and duration given patient-specific characteristics and switching from intravenous formulation to oral [48].

#### Clinical pharmacist

Clinical pharmacists can assist ID specialists or primary team provider by providing evidence-based antifungal recommendations accounting for patient’s clinical characteristics (e.g., renal and hepatic function, mental status, oral intake), drug toxicity, drug-drug interactions, cost, organism susceptibility, and drug availability. They can also monitor therapeutic drug levels and adverse drug events. Education on medication use can be provided by the pharmacists to clinical care teams or to patients. In addition, clinical pharmacists can work with ID specialists to assist in implementing institutional antifungal stewardship program.

#### Pathologist

Identifying fungal elements in diseased tissue is important in establishing a definitive diagnosis of mucormycosis. It is important to note that morphological features alone will not establish a complete diagnosis. Frequent errors in interpreting the mycology reports occur from misidentification of septate and nonseptate organisms, use of inappropriate terminology, and the presence of morphological mimics [49]. Having a pathologist in the multidisciplinary team can increase the chance of accurately identifying the fungal species.

#### Medical microbiologist

Microbiological culture is necessary to establish proven mucormycosis and can provide susceptibility profiles for optimizing therapy [50]. The importance of exchange of information between the clinical team and the microbiologist cannot be over-emphasized [42]. Any clinical suspicion of mucormycosis that the physician may have should be shared with microbiologists. For any critical samples, such as CSF or BAL, the clinical team should appropriately collect and transport samples per institution’s protocol. Any critical results should be reported by the microbiologist over the phone to the caring team. Immediate reporting of the results is the key to initiate appropriate lifesaving treatment promptly.

#### Radiologist

Imaging studies are important to assess the extent of the infection, particularly in ROCM and pulmonary mucormycosis. CT and MRI are recommended in diagnosing mucormycosis. Any findings related to the disease on either study will prompt clinicians to initiate empirical antifungal treatment and additional diagnostic testing.

#### Nursing staff

Since nursing staff spend most time at patient’s bedside among all healthcare providers, they are critical for successfully treating patients with CAM. Having nurses involved in multidisciplinary team discussion can enhance identifying patient’s response to therapy as well as reducing safety issues including medication errors and errors in obtaining and transporting specimens to the laboratory [42]. Furthermore, they can provide patient education to promote self-monitoring of early symptoms and self-hygiene, and to avoid contacts with fungal spores.

## Conclusions

Although CAM is a rare invasive fungal infection, the number of cases is steadily on the rise. While it is difficult to avoid contact with environmental fungal spores, there are strategies to lower the chances of developing mucormycosis. Such strategies include avoiding contact with water-damaged buildings or activities that involve close contact to soil or dust, wearing N95 respirator masks, or wearing long clothing to protect exposed skin [51]. Furthermore, controlling blood glucose levels, following appropriate guidelines for corticosteroid use in treating COVID-19, sanitizing the hospital rooms and linens, and cleaning oxygen tanks are important strategies to reduce the risk of CAM. Upon diagnosis of CAM, antifungal treatment should be initiated promptly along with consideration for surgical interventions. Liposomal amphotericin B is considered the first-line therapy whereas posaconazole or isavuconazole are used as alternative antifungals.

Treating CAM requires a multidisciplinary approach for early diagnosis and prompt initiation of interventions to maximize patient’s chance of survival. By establishing a multidisciplinary team discussion where experts can share their recommendations efficiently and rapidly, this complex and hard-to-treat disease can be best managed. While COVID-19 pandemic continues to pose high level of strain in healthcare system, well-established and efficient multidisciplinary team will be the key to success.

## Data Availability

There is no separate data other than presented in the manuscript.
